# Editorial: Plant microbiome: Ecology, functions, and application trends

**DOI:** 10.3389/fpls.2023.1175556

**Published:** 2023-03-07

**Authors:** Sunil Mundra, Jay Shockey, Mustafa Morsy

**Affiliations:** ^1^ Department of Biology, College of Science, United Arab Emirates University, Al−Ain, Abu−Dhabi, United Arab Emirates; ^2^ Khalifa Center for Genetic Engineering and Biotechnology, United Arab Emirates University, Al−Ain, United Arab Emirates; ^3^ United States Department of Agriculture, Agricultural Research Service, Southern Regional Research Center, New Orleans, LA, United States; ^4^ Department of Biological and Environmental Sciences, University of West Alabama, Livingston, AL, United States

**Keywords:** plant microbiome, microbial ecoclogy, agroecosystem, arbuscular mycorrhizal fungi (AMF), sustainable agriculture, root associated bacteria

The importance of plant- and soil-associated microbiota has been increasingly recognized in recent years. The microbiome is critical in plant development, evolution, ecology, and health, including nutrient uptake, host protection from pathogens, and abiotic stress tolerance. The microbiome community varies across plant compartments, such as soil, roots, and phyllosphere, depending on changes in their physical and chemical characteristics (Singh et al., 2020). Moreover, plant characteristics such as root and shoot morphology and anatomy can change nutrient availability and associated microbiome (Lynch, 2019). Host genetics and environmental factors greatly influence the selection and structure of the plant microbiome (Morella et al., 2020; Shamim et al., 2022). Soil geochemistry, plant genetics, and physiological response significantly influence the root microbiome (Bulgarelli et al., 2012; Lundberg et al., 2012; Schlaeppi et al., 2014; Edwards et al., 2015). Comparable effects of these factors have also been reported on the phyllosphere microbiota of the plant (Vorholt, 2012; Agler et al., 2016; Chen et al., 2020).

Plants’ response to global change depends on their interaction with microbiota. Studying these interactions can help develop strategies for managing soil health and combating plant diseases, which will improve crop productivity and sustainability. This Research Topic delves into various plant microbiota studies, which offer insights into the complex interactions between plants and their associated microbiota. Overall, understanding plant microbiota is crucial for developing sustainable agriculture and addressing the challenges of global change. By expanding our understanding of the complex interactions between plants and their associated microbiota, we can develop innovative solutions to enhance crop productivity, soil health, and environmental sustainability.

The microbiome of natural ecosystems, such as agroecosystems, is constantly changing due to various biotic and abiotic factors. Previous research has shown the impact of soil chemistry, such as salinity, on bacterial communities in desert soil (O’Brien et al., 2019; Nan et al., 2022), but little is known about the effects of irrigation water characteristics, such as salinity and pH, on the microbiota. Understanding the impact of environmental factors, such as irrigation water characteristics, on microbiota in agroecosystems is crucial for developing sustainable agriculture practices. One study by Loganathachetti et al. has focused on the impact of saline groundwater irrigation on bacterial communities in date palm cultivated soil. The study found that water salinity had a distinct selection effect on bacterial communities and increased the abundance of specific bacteria, such as Mycobacterium-saline groundwater and Subgroup_10-freshwater irrigation, between irrigation water sources in date palm cultivated soil. These bacterial groups could potentially play a role in nutrient cycling despite the harsh environmental conditions of arid agroecosystems.

Fungal diseases caused by plant pathogens pose a significant challenge to agroecosystems. The widespread use of fungicides for disease control has resulted in developing fungal resistance (Müller et al., 2021) and the accumulation of chemical residues in seeds (Wei et al., 2022). For instance, soybean crops suffer from significant productivity loss due to leaf rust disease caused by *Phakopsora pachyrhizi*. In this regard, biocontrol candidates can provide an eco-friendly alternative for disease management. In the current Research Topic, Twizeyimana et al. conducted a study to screen 998 bacterial strains for biocontrol of leaf rust in soybean. The study identified six biocontrol candidates from the Bacillus and Pseudomonas genera, which showed effective disease suppression in the detached leaf, greenhouse, and field trials. Furthermore, the study demonstrated a significant correlation between the outcomes of the detached leaf assay and those of the greenhouse and field trials. This finding indicates that the detached leaf assay can be a rapid screening tool for identifying potential biocontrol microbes in future studies.

Arbuscular Mycorrhizal Fungi (AMF) are critical to plant growth and resilience to environmental stresses. They form symbiotic relationships with the roots of most plants, where the length of AMF colonization within the root system is vital for nutrient uptake and overall plant growth (Barceló et al., 2020). To enhance plant productivity and reduce dependence on chemical fertilizers, it is crucial to understand factors that influence AMF-driven plant growth promotion, such as experimental conditions, plant type, and root-shoot ratio. In this Research Topic, Qin et al. screened 639 published articles with 1,640 observations. Their meta-analyses revealed that experimental duration and pot size were the most significant factors influencing AMF-driven plant growth promotion-related traits, such as plant shoot, root, and total biomass. Plant functional type and guilds also significantly impacted shoot and root biomass, respectively. The study also emphasizes the need to consider plant type and root-shoot ratio, which can vary widely across plant species, in AMF research. This information could help optimize the use of AMF in plant growth promotion, particularly in agroecosystems where chemical fertilizers can negatively impact soil health and the environment.

Understanding root phenomics and the complex interactions between roots and the environment is critical for improving crop productivity and sustainability. Root phenotypes, including anatomy, branching, architecture, and depth, are vital in nutrient uptake and microbiome composition. Birt et al. summarized the different root phenotypes and highlighted their significance in altering nutrient availability and the microbiome. One important recommendation of the mini-review was the development of recombinants capable of supporting desired microbiomes. Additionally, understanding phenotypic diversity between ancestral and domesticated varieties and characterizing fine-scale changes in the rhizosphere are essential research priorities. These research areas will enable us to identify novel strategies for enhancing plant-microbe interactions in diverse environmental conditions, ultimately improving crop yields, resilience, and sustainability.

The articles published in this Research Topic address critical knowledge gaps in plant microbiomes, particularly in extreme environments. Studies focused on microbial communities in arid agroecosystems and their response to salinity and irrigation water quality, as well as biocontrol candidates for managing fungal diseases, provide insights into the complex interactions between plants and microbes. Furthermore, meta-analyses examining the effects of experimental conditions and plant traits on the role of AMF in plant growth promotion shed light on the importance of plant-microbe interactions in sustainable agriculture. These studies demonstrate plant-microbe interactions’ potential in enhancing crop productivity and sustainability in diverse environmental conditions.

## Author contributions

All authors made a substantial, direct, and intellectual contribution to the work and approved it for publication.
